# Medications in workplace – a literature review

**DOI:** 10.1080/07853890.2021.1896846

**Published:** 2021-09-28

**Authors:** Catarina Egreja, Noémia Lopes

**Affiliations:** ^a^Centro de Investigação Interdisciplinar Egas Moniz (CiiEM), Egas Moniz Cooperativa de Ensino Superior, Caparica, Portugal

## Abstract

**Introduction:**

The purpose of this paper is to present the main conclusions drawn from a literature review on the theme "Medications in Workplace". The interest that led to this literature collection is based on a larger project, currently underway,^1^ that focuses on the use of medicines, food supplements and other natural products to improve physical, intellectual and social performance (performance consumption [1]) in three professional groups. With this presentation we seek to highlight the correlation between aspects of the nature of work and the consumption of these substances, the motives associated with it and the predominance of uses.

**Materials and methods:**

The proposed presentation is characterised as a theoretical essay, based on a review of the literature on the topic “Medications in Workplace”, performed through an extensive bibliographical search on specialised online platforms with a peer-review policy, following predetermined search terms, that resulted in the collection and analysis of over a dozen of scientific articles, reports from governamental agencies and monographs, on the fields of social sciences and medicine. Such studies were carried out in different countries over an extended period of time (1990-2018) and focussed on professional groups such as nurses, office workers and teachers.

**Results:**

The main aspects of work associated with substance use are stress, shift work and night time work, mainly because of their impact on the quality of sleep [2]. The management of fatigue (physical and mental) and of the ability to concentrate in order to improve work performance is carried out, in several cases, through the consumption of certain substances [3]. These range from caffeine, to medicines (taken with or without a prescription), or even illegal drugs. While the reasons for consumption are quite homogeneous, its frequency varies strongly between studies due to different methodologies and conceptual criteria used.

**Discussion and conclusions:**

The difficult quantification of consumption does not preclude the conclusion that we are dealing with a socially complex phenomenon when we speak of performance enhancing consumption that shows a change in the conventional use of therapeutic resources beyond the frontiers of health and disease that is important to continue studying, particularly from its social contexts. For instance, to analyse if there are professional groups particularly vulnerable to these auxiliary consumptions and which factors differentiate them.
